# Myeloid C-Type Lectin Receptors in Tuberculosis and HIV Immunity: Insights Into Co-infection?

**DOI:** 10.3389/fcimb.2020.00263

**Published:** 2020-06-03

**Authors:** Kubra F. Naqvi, Janice J. Endsley

**Affiliations:** Department of Microbiology and Immunology, The University of Texas Medical Branch, Galveston, TX, United States

**Keywords:** tuberculosis, HIV, TB and HIV co-infection, innate immunity, C-type lectin receptors

## Abstract

C-type lectin receptors (CLRs) are carbohydrate binding pattern recognition receptors (PRRs) which play a central role in host recognition of pathogenic microorganisms. Signaling through CLRs displayed on antigen presenting cells dictates important innate and adaptive immune responses. Several pathogens have evolved mechanisms to exploit the receptors or signaling pathways of the CLR system to gain entry or propagate in host cells. CLR responses to high priority pathogens such as *Mycobacterium tuberculosis (Mtb)*, HIV, Ebola, and others are described and considered potential avenues for therapeutic intervention. *Mtb* and HIV are the leading causes of death due to infectious disease and have a synergistic relationship that further promotes aggressive disease in co-infected persons. Immune recognition through CLRs and other PRRs are important determinants of disease outcomes for both TB and HIV. Investigations of CLR responses to *Mtb* and HIV, to date, have primarily focused on single infection outcomes and do not account for the potential effects of co-infection. This review will focus on CLRs recognition of *Mtb* and HIV motifs. We will describe their respective roles in protective immunity and immune evasion or exploitation, as well as their potential as genetic determinants of disease susceptibility, and as avenues for development of therapeutic interventions. The potential convergence of CLR-driven responses of the innate and adaptive immune systems in the setting of *Mtb* and HIV co-infection will further be discussed relevant to disease pathogenesis and development of clinical interventions.

## Introduction

TB and HIV are the leading causes of death by infectious agents globally (WHO, 2019a,b). In 2018, an estimated 10 million people fell ill with TB and 1.2 million deaths occurred among HIV-negative people globally (WHO, 2019a). Approximately 37.9 million people are currently living with HIV/AIDS and 1.1 million die each year (WHO, 2019b). Tuberculosis remains a large risk factor for people living with HIV (PLWH)/AIDS and HIV-associated TB was the cause of an additional 251,000 deaths in 2018. The co-infection of *Mycobacterium tuberculosis* (*Mtb)* and HIV contributes to the large burden on healthcare systems of endemic areas and have increased the priority of improved co-infection therapeutic strategies (WHO, 2019a,b).

Cell mediated immunity (CMI), especially that mediated by CD4^+^T cells, is essential for host resistance to *Mtb* (Cooper, 2009). HIV infection drives progressive depletion and dysfunction of leukocytes of the CMI response (Pawlowski et al., 2012) including defects that persist following anti-retroviral therapy (ART). A synergistic deterioration of the immune system is associated with aggressive disease in those with co-infection through mechanisms that are incompletely characterized. Although the loss of protective immunity in PLWH is primarily attributed to CD4^+^T cell loss and dysfunction in the progression to AIDS, those with co-infection nonetheless display immune disturbance prior to significant T cell loss (Sharma et al., 2005; de Noronha et al., 2008; Sester et al., 2010).

Innate and adaptive immune cells which are resistant to direct infection display functional defects in the setting of HIV infection due to indirect effects on cell toxicity and immune signaling networks by viral mediators (Mazzuca et al., 2016; Garg and Joshi, 2017). Myeloid cell populations including monocytes, macrophages and dendritic cells are targets for HIV infection; macrophages in particular are an important viral reservoir (Igarashi et al., 2001; Heesters et al., 2015; Honeycutt et al., 2017). As the primary hosts for *Mtb* propagation, the direct and indirect effects of HIV infection on myeloid cell innate function are an important and poorly understood factor for the outcome of co-infection.

Signaling through myeloid cell PRRs dictates important innate recognition and responses to *Mtb* and HIV molecular patterns that may direct the progression of disease in co-infection scenarios. Engagement of PRRs such as toll-like receptors (TLR), nod-like receptors (NLR) and CLRs, and the downstream immune responses that are elicited is critical for dictating outcomes of individual disease during TB or HIV as previously reviewed (Mesman and Geijtenbeek, 2012; Hossain and Norazmi, 2013; Mortaz et al., 2015). In brief, TLRs interact with specific mycobacterial ligands to initiate phagosome maturation, pro-inflammatory, and anti-inflammatory cytokine secretion (e.g., IL-12, IL-18, and IL-10) (Kim et al., 2019). The resulting induction of IFN-γ further activates antimicrobial pathways of infected macrophages (Hossain and Norazmi, 2013; Mortaz et al., 2015). HIV infection activates TLR signaling pathways (Meier et al., 2007; Lester et al., 2008) that contribute to host restriction through induction of antiviral interferons or activation of cytokines that promote viral transcription (Mesman and Geijtenbeek, 2012).

Nod-like receptors are cytosolic PRRs which recognize intracellular bacterial and viral pathogens to further mediate macrophage activation. NOD-1 and 2 signaling following *Mtb* infection facilitates mycobacterial survival by suppressing apoptosis of macrophages (Mortaz et al., 2015). Additionally, NLRP3-dependent inflammasome activation of macrophages exposed *in vitro* to *Mtb* promotes IL-1β secretion and cell death (Mortaz et al., 2015). Inflammasome activation by NOD-like receptors is also a major mechanism of pyroptotic CD4^+^T cell depletion due to HIV in *ex vivo* models of infection (Doitsh et al., 2014; Tomalka et al., 2016). HIV genomic RNA and newly synthesized mRNA in infected macrophages is also detected by the Rig-I like receptors (RLR) in the cytosol. RLRs drive the production of type-1 IFNs following recognition of intracellular pathogens (Bergantz et al., 2019), inducing expression of interferon stimulated genes and eliciting an antiviral response. However, HIV can evade this mechanism through degradation of RIG-I by a viral protease (Bergantz et al., 2019).

The surface bound CLRs, especially abundant on innate leukocytes of the myeloid lineage, also bind to molecular patterns of *Mtb* and HIV (Turville et al., 2003; Mishra et al., 2017). The characteristic glycosylation of the mycobacterial cell wall and HIV envelope protein gp120 (Turville et al., 2001; Guttman et al., 2015; Ishikawa et al., 2017), engage CLRs, which recognize carbohydrate motifs. Molecular recognition through CLRs triggers a myriad of downstream signaling events that modulate ligand and CLR-specific immune outcomes (Geijtenbeek and Gringhuis, 2009). Our understanding of the role of CLRs in TB and HIV mono-infections is rapidly evolving through active investigation. In contrast, the effect of co-infection to exacerbate, confound, or otherwise perturb signaling through CLR pathways is largely uncharacterized.

This review will discuss the current state of knowledge regarding CLRs with described roles in innate immune recognition of *Mtb* and HIV. The contribution of these CLR pathways to protective and non-protective immune outcomes will be discussed in the context of mono- and or co-infection settings. Finally, the potential to exploit CLR pathways for clinical interventions that prevent or reduce disease due to this important dual pandemic will be discussed.

## CLRs Regulate Protective and Pathogenic Outcomes of TB and HIV

Dendritic cells (DCs) and macrophages are sentinel antigen presenting cells (APC) and are therefore well equipped with PRRs to engage molecular motifs of *Mtb* or HIV during infection. The CLRs of antigen presenting cells bind self and non-self-antigens to activate and/or suppress immune function and internalize pathogens (McGreal et al., 2005). CLRs can be classified based on structural, carbohydrate recognition and signaling features (Dambuza and Brown, 2015). The carbohydrate recognition domain is a compact structural motif of CLRs which determines carbohydrate specificity and binds ligands in Ca^2+^ dependent or independent manner (Geijtenbeek and Gringhuis, 2009).

**Figure 1:**
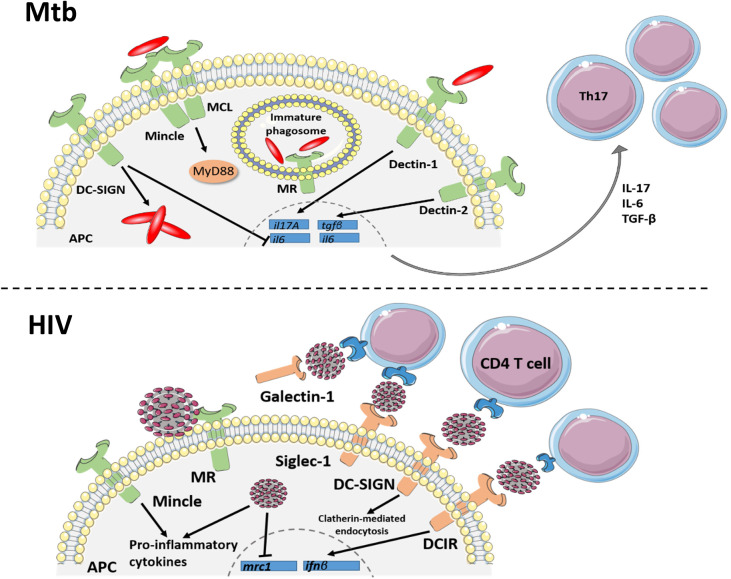
C-type lectin receptor signaling in TB and HIV. Signaling through C-type lectins is an important driver of innate and adaptive responses against *Mtb* infection. Dectin 1 and 2 signaling within APCs results in expression of *il17A, il6* and *tgf*β transcript for the differentiation of Th17 cell subsets. DC-SIGN can inhibit the IL-6 response of Dectin-1, producing an anti-inflammatory environment, which favors *Mtb* intracellular growth. Mincle, which binds to mycobacterial TDM, forms a heterodimer with another CLR, MCL, to induce protective cytokine responses through the MyD88 signaling pathway. Mannose receptor (MR), binding to mannosylated *Mtb*, initiates phagocytosis while also inhibiting phagosome maturation within macrophages. Additionally, C-type lectin receptors bind to the HIV envelope protein, gp120, to mediate or restrict viral transmission and regulate inflammatory responses. The transmembrane CLRs, DC-SIGN, DCIR and siglec-1 facilitate trans-infection of HIV to CD4^+^T cells through the lymphatics. Additionally, the soluble CLR, galectin-1, improves HIV binding to the CD4 receptor. Signaling of dendritic cell receptors, DC-SIGN and DCIR, also aid in host defense against HIV by internalizing viral particles and initiating an antiviral type I interferon response, respectively. As another mechanism of host defense, the macrophage mannose receptor restricts viral particle budding, although HIV can evade this mechanism by downregulating the *mrc1* gene. HIV also increases Mincle-induced pro-inflammatory cytokine production in *in vitro* models of infection. Together, these CLRs demonstrate the multifaceted role of receptor binding in HIV protection and pathogenesis and regulating the innate immune response to *Mtb*.

The transmembrane CLRs are further divided into groups, the type I mannose receptor family and the type II asialoglycoprotein receptor family which includes further subfamilies (dectin 1 and DCIR) (Geijtenbeek and Gringhuis, 2009). The signaling motifs of CLRs can also be used to categorize these receptors further. Transmembrane CLRs can include immunoreceptor tyrosine-based activation (ITAM) or inhibitory (ITIM) motifs while some CLRs signal through a single tyrosine-based motif termed hem-ITAM (Osorio and Reis e Sousa, 2011). Many transmembrane and soluble CLRs carry out essential functions in immune homeostasis and innate responses against a wide variety of pathogens. This review will focus on CLRs associated with *Mtb* and HIV infections including the mannose receptor, Mincle, Dectins 1 and 2, Mannose Binding Lectin, DC-SIGN, Langerin and DCIR (Figure 1).

## Mannose Receptor

The mannose receptor (MR, CD206) is a type I transmembrane CLR present on alveolar macrophages, M2 macrophages and dendritic cells. Binding of MR to sulfated and mannosylated sugars results in internalization of antigen and delivery to the MHC II and CD1b pathways (Martinez-Pomares, 2012). Receptor engagement by *Mtb* has served as a model system to characterize MR signaling outcomes and further define an innate immune role in TB. Virulent *Mtb* induces PPAR-γ-dependent inflammatory responses, including IL-8 and cyclooxygenase 2 expression, through a MR dependent pathway (Rajaram et al., 2010). MR binds to *Mtb* liparabinomannan and mannosylated proteins, resulting in interaction with the FcRγ-chain which mediates surface localization and activation of the Grb2 adaptor molecule required for phagocytosis (Rajaram et al., 2017). MR recruitment of the tyrosine phosphatase, SHP-1, however, limits the PI(3)P production needed for maturation of the *Mtb-*containing phagosome (Rajaram et al., 2017). As currently understood, recognition of *Mtb* PAMPs through MR activates important inflammatory and phagocytic function of APCs, but may also promote intracellular *Mtb* survival through inhibition of phagosome-lysosome fusion.

The distribution of MR as a phenotypic marker of alternatively activated, or M2, macrophages in unique subsets and disease processes has also informed our understanding of its immune role. MR is a selective marker of perivascular macrophages in human and non-human primate CNS tissue and a marker of phenotypic switch (CD206+ to CD206-) during HIV neuroinflammation (Holder et al., 2014). Expression of MR on peripheral macrophages can also serve as a potential biomarker to identify those with dual HIV strain infections. MR expression decreases following ART treatment for patients with HIV-1 or HIV-2, and remains constant in those with dual infection despite treatment (Andersen et al., 2018). Cellular expression of MR also contributes to sexual transmission of HIV as an alternative entry receptor in vaginal epithelial cells, the primary site of male-to-female transmission (Jadhav et al., 2013).

Binding of MR to HIV gp120 induces production of matrix metalloproteinase (MMPs) breaking down the extracellular matrix and permitting HIV to cross the epithelial barrier to gain access to sub-epithelial CD4^+^ cells (Fanibunda et al., 2011). In addition to expression on vaginal epithelial cells, MR expression on sperm cells has been shown *in vitro* to mediate infection to susceptible cells, suggesting a possible role for sperm cells as vectors for sexual transmission of HIV (Fanibunda et al., 2008; Cardona-Maya et al., 2011). Binding of HIV gp120 to MR is further enhanced by oligomerization of the receptor on macrophages and dendritic cells, further promoting trans-infection of CD4^+^T lymphocytes (Lai et al., 2009). An anti-viral role for MR has also been described and occurs through prevention of HIV particle release from infected cells (Sukegawa et al., 2018). Interestingly, HIV counteracts this antiviral activity by repressing MR transcription (Sukegawa et al., 2018).

The role for MR in the innate function of APCs in response to *Mtb* or HIV is increasingly understood to be complex; activating important antimicrobial mechanisms and serving as an avenue for immune exploitation. To date, studies in the setting of experimental co-infection that would show how simultaneous or synergistic activation through MR signaling could impact myeloid cell function are lacking. These are important given the potential for co-infection signaling events to influence disease processes. Speculatively, the *Mtb*-driven MR signaling of inflammation could augment viral replication driven through cytokine pathways known to activate the HIV promoter (Jiang and Dandekar, 2015). Similarly, activation of MMPs by HIV signaling through MR could affect the development and maintenance of *Mtb* granulomas which are important for mycobacterial containment (Al Shammari et al., 2015; Sabir et al., 2019).

## Mincle

Macrophage-inducible C-type lectin (Mincle, Clec4e), is a type II transmembrane CLR expressed by myeloid cells, such as macrophages, and contains a single carbohydrate recognition domain. Mincle binds both foreign and self-antigens including motifs found on fungal and bacterial surfaces and damaged-self antigens including SAP130 and β-glucosylceramide that are released from necrotic cells (Miyake et al., 2010; Nagata et al., 2017). Binding of Mincle to fungal pathogens such as *C. albicans* and Malassezia species is an important component of anti-fungal immunity mediated through TNF-α induction. The binding of Mincle to trehalose-6,6'-dimycolate (TDM) of the mycobacterial membrane activates macrophages to produce nitric oxide(NO) and inflammatory cytokines that contribute to antibacterial function and granuloma formation, respectively (Miyake et al., 2010). Consistent with other CLRs, Mincle deficiency does not alter the disease outcome in experimental murine TB. The inflammatory response that occurs upon *in vitro* binding of Mincle by TDM (Lang, 2013) is also fully compensated for *in vivo* (Heitmann et al., 2013).

In contrast to Mincle, the macrophage C-type lectin (MCL) co-receptor for Mincle has been shown to be indispensable for protective TB immunity (Wilson et al., 2015). MCL^−/−^ mice exhibit increased mortality, bacterial burden and pulmonary inflammation following *Mtb* infection, compared to WT. MCL shares high AA sequence homology with Mincle and is also expressed by myeloid populations (Miyake et al., 2013, 2015). Mincle expression is positively regulated by MCL, an outcome postulated to reflect the heteromeric complex formation of Mincle and MCL (Miyake et al., 2015). Formation of the Mincle/MCL complex occurs downstream of Mincle-dependent MyD88 signaling and contributes to protective anti-mycobacterial responses (Kerscher et al., 2016).

A role for Mincle in HIV disease outcomes has not been described to date. Similarly, the contribution of Mincle signaling to co-infection outcomes is poorly understood due to a lack of knowledge related to HIV activation and paucity of investigations of experimental co-infections. A recent study, however, suggested that peripheral blood myeloid cells from HIV+ subjects respond differently to *Mtb* Mincle agonists (Zapata et al., 2019). Interestingly, *in vitro* activation of Mincle using a TDM analog promoted increased pro-inflammatory responses by peripheral blood monocytes from HIV+ and aged subjects (Zapata et al., 2019). These results suggest that HIV infection may disturb Mincle signaling in the setting of co-infection with *Mtb* and potentially alter immune outcomes.

## Dectin 1

The type II transmembrane CLR, Dectin-1, is expressed on myeloid cells including monocytes, macrophages, dendritic cells and neutrophils, and binds to β-glucans commonly found on fungal cell walls (Tsoni and Brown, 2008). Ligand binding of Dectin-1 induces a downstream signaling response which results in phagocytosis, respiratory burst, and a pro-inflammatory response that promotes fungal clearance (Brown, 2006; Dennehy and Brown, 2007). In addition to regulating innate immune responses to pathogens, Dectin-1-induced cytokines modulate subsequent adaptive responses (Brown, 2006). Blocking Dectin-1 signaling in a human PBMC model of *in vitro Mtb* infection reduced production of IL-17A through a mechanism that required co-signaling with TLR4 and endogenous IL-1 (van de Veerdonk et al., 2010). The IL-17 producing helper T cells (Th17) are among the essential CD4^+^T cell subsets that direct anti-mycobacterial responses of the adaptive immune system. The Dectin-1 pathway could thus be exploited as a novel vaccination strategy to activate Th17 cell memory to *Mtb* and other important pathogens that rely on IL-17 for protective immunity (Scriba et al., 2008).

Similar to Mincle, the immune functions activated through Dectin-1 appear to have redundant mechanisms, as Dectin 1- deficient mice display similar immune response and pulmonary disease as wild type counterparts (Marakalala et al., 2011). Deficiency of a single signaling intermediate rarely results in markedly increased pulmonary TB. An exception is deficiency of CARD9, the central adaptor for many CLRs including both Mincle and Dectin-1. *Mtb*-infected mice lacking CARD9 display severe pyogenic and neutrophilic pneumonia, and fatal TB disease (Dorhoi et al., 2010). Interestingly, deficiency in CARD9 did not impair the *in vitro* antimicrobial activity of macrophages and did not alter Th17 or other T cell responses *in vivo* (Dorhoi et al., 2010). The substantial influx of neutrophils observed in *Card9*^−/−^ mice is consistent with observations that loss of signaling through MCL or CTLR (Clec9a) compromises the regulation of IL-1 and CXCL8 responses of *Mtb*-infected macrophages (Wilson et al., 2015; Cheng et al., 2017).

In contrast to the activation of protective immune responses to *Mtb*, HIV engagement of Dectin-1 promotes viral replication by facilitating *cis-*infection of immature DCs (Cote et al., 2013). Dectin-1 engagement by opportunistic pathogens can also promote viral pathogenesis. Binding of Dectin-1 by β-glucans of fungal pathogens such as histoplasmosis results in reduced leukotriene B4 levels (Sorgi et al., 2009). An imbalance between leukotrienes and prostaglandins favors HIV proliferation and establishment of opportunistic infection due to the role of PGE2 in inhibiting microbial killing (Sorgi et al., 2009). The balance between leukotrienes and prostaglandins also influences survival in experimental models of TB and are identified as a correlate of TB-diabetes co-morbidity (Tobin et al., 2012; Mayer-Barber et al., 2014; Shivakoti et al., 2019).

## Dectin-2

Dectin-2, present on tissue macrophages, some DC subsets including Langerhans cells, and peripheral blood monocytes, recognizes numerous fungal and bacterial pathogens (Graham and Brown, 2009). Dectin-2 binds zymosan of fungal species such as *Candida albicans* and mannose rich glycolipids such as lipoarabinomannan (Man-LAM) of *Mtb* (Decout et al., 2018). Binding of Dectin-2 to Man-LAM activates pro-inflammatory (IL-6 and TNF-α) and immune regulatory (IL-10 and TGF- β) cytokines (Yonekawa et al., 2014). Consistent with the roles of IL-6 and TGF- β to promote Th17 differentiation, binding of Man-LAM by Dectin-2 drives generation of *Mtb*-specific Th17 cells (Decout et al., 2018).

Although other Dectin-2 family CLRs, such as DCIR, are associated with HIV transmission, a role for Dectin-2 signaling in HIV mono-infection and co-infections with *Mtb* has not been described. The function of both Dectin 1 and 2 to promote differentiation of Th17 cells during the adaptive response to other pathogens such as *Mtb*, however, may have consequences in the setting of HIV co-infection. Activated CD4^+^T cells with a memory phenotype are more susceptible to HIV infection and support greater viral replication than resting T cell counterparts. Among various CD4^+^T cell populations, the Th17 populations are especially susceptible to HIV infection, lacking inhibitory RNAses that limit viral replication in other cells (Sun et al., 2015; Christensen-Quick et al., 2016; Lee and Lichterfeld, 2016; Fernandes et al., 2017). Th17 cells expressing CCR6 were recently described to function as a long lived HIV reservoir that persists in PLWH despite ART pressure (Sun et al., 2015; Fernandes et al., 2017; Gosselin et al., 2017). CLR-dependent pathways that promote differentiation of *Mtb*-specific Th17 cells may thus contribute to HIV proliferation and persistence. Depletion and compromise of Th17 cells due to HIV infection could similarly compromise protective function of the cell mediated immune response to *Mtb*.

## Mannose Binding Lectin

Mannose binding lectin (MBL) belongs to a family of secreted proteins known as collectins and primarily circulates through serum, synovial and amniotic fluid. MBL ligand binding is Ca^2+^ dependent and selective for terminal mannose, fucose and N-acetylgalactosamine (GalNAC)(Ip et al., 2009). Due to the broad ligand binding and presence in the serum, MBL is an important receptor for opsonization of pathogens to clear infection and prevent recurrent infections (Ip et al., 2009). MBL binding to *Mycobacterium avium* was previously reported (Polotsky et al., 1997), while recent studies have demonstrated a broader role in mycobacterial immunity. Ligation of MBL to *Mtb* and the related *M. bovis*, results in enhanced phagocytosis, lectin pathway activation and agglutination of bacteria (Bartlomiejczyk et al., 2014). Additionally, binding of MBL to *Mtb* activates MASP1 homodimers to catalyze activation of MASP2 and ultimately generation of C3 convertase (Klassert et al., 2018) in the immune complement cascade. The function and expression of MBL is dependent on polymorphisms in the *MBL2* gene, which encodes the receptor. Polymorphisms in *MBL2* are associated with susceptibility to pulmonary and extra-pulmonary TB in various human populations (da Cruz et al., 2013; Nisihara et al., 2018).

The HIV envelope displays several conserved glycan residues that are epitopes for broadly neutralizing antibodies (bnAb) produced by B cells of the acquired immune system. MBL present in human serum is able to block binding of the 2G12 bnAb to HIV, and as a result, increase virus infectivity (Marzi et al., 2007). Serum concentrations of MBL may thus limit the efficacy of neutralizing antibody-based prevention or treatment strategies for HIV. The outcome of MBL signaling in the setting of co-infection has not been described to date. Activation of MBL by *Mtb* exposure could be explored as a potential mechanism for the increased viral replication observed in those with dual infection. Similarly, increased HIV replication due to inhibition of bnAb by MBL could further compromise CMI to *Mtb*.

## Dc-Sign

The CLR which has been most extensively studied in *Mtb* and HIV infections is DC-SIGN. This mannose-binding type II CLR is present on dermal DCs, interstitial DCs of the mucosa, and macrophages (van Kooyk and Geijtenbeek, 2003). DC-SIGN interacts with the LPS structure of *Klebsiella pneumonia* and *Helicobacter pylori* to internalize and target these pathogens for antigen presentation (van Kooyk and Geijtenbeek, 2003). In experimental TB, selective knockout of the murine homolog for human DC-SIGN (Tanne and Neyrolles, 2010), SIGNR1, only partially impairs host resistance to *Mtb*. However, deficiency in both SIGNR1 and murine MR results in increased lung inflammation and larger foci of bacilli 5 months post-infection (Court et al., 2010). These disease outcomes are moderate, however, compared to those in mice lacking the essential cytokine pathways (TNF, IL-1 or IFN-γ) which lack compensatory mechanisms and are unable to control *Mtb* (Court et al., 2010).

Although DC-SIGN is primarily expressed on dendritic cells, expression on immunoregulatory macrophages has been shown in the pleural cavity of human patients and lung tissue from NHPs with active TB (Lugo-Villarino et al., 2018). The functional outcome of DC-SIGN signaling within *Mtb*-exposed myeloid cells remains to be fully characterized, however, modulation of the Dectin-1 driven pro-inflammatory responses has been observed in a subset of M2 macrophages (Lugo-Villarino et al., 2018). Further investigations are required to determine if DC-SIGN pathways may work in concert with other CLRs or PRRs to regulate damaging pro-inflammatory responses to *Mtb*.

DC-SIGN also serves as an entry receptor for several viral pathogens including Dengue virus, Ebola virus, hepatitis C virus and cytomegalovirus (van Kooyk and Geijtenbeek, 2003). A role for DC-SIGN in trans-infection with HIV and the closely related simian immunodeficiency virus (SIV) has been well-documented in human and non-human primate cells, respectively (Geijtenbeek et al., 2001). Adhesion of gp120 to DC-SIGN promotes viral transmission via the lymphatics while expression of DC-SIGN by placental Hofbauer cells permits vertical transmission of HIV (Soilleux et al., 2001; da Silva R. C. et al., 2011). Expression of DC-SIGN has also been described on HIV-susceptible tissues such as colonic mucosa (Preza et al., 2014) and foreskin epithelium (Hirbod et al., 2010) as a potential mechanism of HIV transmission to and from those sites.

The function of DC-SIGN in viral infection and transmission highlights the specificity and overlap of the CLR interactions with HIV. Although DC-SIGN promotes trans-infection of HIV, many other CLRs carry out a similar function independent of DC-SIGN. As previously discussed, MR facilitates HIV transmission by binding to mannose moieties of gp120 through a mechanism inhibited by pre-incubation of cells with mannan (Nguyen and Hildreth, 2003). The heavy glycosylation of HIV gp120 also contains sialic acid residues which are recognized by siglecs. These sialic acid binding lectins, present on macrophages and DCs, facilitate viral adhesion and infection of R5-tropic viruses to macrophages (Zou et al., 2011). In particular, siglec-1 expressed on mature DCs of the cervical mucosa enhances viral uptake and trans-infection, thus enabling systemic viral spread (Izquierdo-Useros et al., 2014; Perez-Zsolt et al., 2019).

Soluble CLRs, such as galectin-1, are exploited by HIV to enhance binding of gp120 and the CD4 receptor to facilitate viral infection of T cells (St-Pierre et al., 2011). CLRs which bind to HIV gp120 are also able to competitively inhibit viral binding to DC-SIGN and thus, block trans-infection. The CLR surfactant protein SP-D, present in mucosal tissue, inhibits viral entry and DC-SIGN interaction by competitively binding HIV gp120 (Dodagatta-Marri et al., 2017). In short, CLR binding to gp120 and other carbohydrate rich molecular motifs is an important mechanism for HIV pathogenesis as well as a potential avenue for clinical intervention.

The association of DC-SIGN in immune evasion strategies employed by *Mtb* and HIV has been characterized as previously reviewed (Kaufmann and Schaible, 2003; van Kooyk et al., 2003). In brief, during co-infection with *Mtb*, DC-SIGN mediated Raf-1 signaling has been shown to be critical for HIV transcription elongation and can even enhance viral replication (Gringhuis et al., 2010). Additional studies of the antigen uptake mechanism of DC-SIGN have identified the involvement of membrane cholesterol and dynamin for endocytosis and the clatherin-dependent HIV internalization into dendritic cells (Cambi et al., 2009).

## Langerin

The CLR Langerin is specifically expressed by Langerhans immature DC of the epidermis and mucosal tissues (de Witte et al., 2008). Langerin is a type II transmembrane receptor which binds mannose, fucose, and GlcNAC in a Ca^2+^ dependent manner (van der Vlist and Geijtenbeek, 2010). As a consequence of broad spectrum carbohydrate recognition, Langerin recognizes a number of PAMPs including those expressed by *Candida albicans* and *Mycobacterium leprae*. To date, the subsequent downstream signaling events triggered by Langerin binding are poorly characterized. Additionally, although it is likely that Langerin recognizes components of the glycosylated *Mtb* cell wall, direct binding has not been described (van der Vlist and Geijtenbeek, 2010).

Langerhans cells are among the first innate immune cells to encounter mucosal pathogens and are therefore important gatekeepers to prevent infection at those sites (van der Vlist and Geijtenbeek, 2010). To that effect, binding of Langerin to HIV initiates a TRIM5α dependent pathway which targets HIV for autophagic degradation (Ribeiro et al., 2016). Interestingly, DC-SIGN engagement abrogates this restriction pathway by causing a dissociation of TRIM5α from the receptor and thus, permitting infection (Ribeiro et al., 2016). These findings highlight the synergistic ability of DC CLRs to dictate the outcome of HIV infection by permitting or restricting pathogen replication and immune responses. Additionally, therapeutic strategies which block DC-SIGN may in turn augment the protective function of Langerin signaling during HIV infection. Identification of *Mtb* binding by Langerin could suggest avenues whereby Langerin and DC-SIGN signaling convergence, or interference, may impact immune outcomes in co-infected individuals.

## Dcir

Dendritic cell immunoreceptor (DCIR) is highly expressed on peripheral blood leukocytes including monocyte-derived DCs, macrophages, neutrophils, and plasmacytoid DCs (Graham and Brown, 2009). DCIR contains an immunoreceptor tyrosine-based inhibition motif (ITIM) associated with downregulation of pro-inflammatory signaling. Similar to other related CLRs, DCIR-dependent internalization targets antigens for presentation on APCs, thus promoting T-cell proliferation (Graham and Brown, 2009). As mentioned previously, the DCIR related CLR, Dectin-2, promotes Th17 responses upon engagement with *Mtb* antigen (Decout et al., 2018). Similarly, recognition of *Mtb* by DCIR promotes Th17 T cell differentiation while also activating the IFNR-associated JAK-STAT pathway to sustain type I IFN signaling and reduce Th1 responses. Expectedly, knockout of DCIR improves *Mtb* control but also results in increased pulmonary pathology (Troegeler et al., 2017). More interesting, however, is the immune activating nature of DCIR in TB immunity which is unexpected for an ITIM-containing CLR, although this mechanism is yet to be determined (Troegeler et al., 2017).

Analogous to DC-SIGN, DCIR plays an important role in promoting HIV viral capture and replication within CD4^+^T cells through activating phosphorylation of the receptor ITIM domain (Lambert et al., 2008, 2011). Blocking of DCIR has been employed as a therapeutic strategy to prevent HIV transmission and may be an effective strategy to improve Th1 responses against *Mtb* in a co-infection scenario. The role of DCIR to promote Th17 differentiation, similar to Dectin 1 and 2, may increase viral replication and persistence due to the susceptibility of Th17 cells to HIV.

## Genetic Polymorphisms of ClRs as Disease Predictors

Polymorphisms of PRR genes within the human population have been increasingly associated with disease resistance and susceptibility. Functional polymorphisms in CLR genes that result in receptor impairment have been linked to increased susceptibility to infections such as invasive pneumonia and hepatitis (Eisen and Minchinton, 2003), as well as chronic diseases such as Crohn's disease (Marquez et al., 2009). The population diversity of several CLRs, especially MR, Mincle, and MBL, has been further linked to increased susceptibility to HIV and *Mtb* within endemic areas through diverse mechanisms including: modulation of immune outcomes, improved pathogen transmission, and associated co-morbidities.

Recognition of Man-LAM by MR plays a key role in innate immunity to *Mtb* (Kang et al., 2005). A single nucleotide polymorphism (SNP) of the *MRC1* gene which encodes the MR was recently identified as a candidate for genetic susceptibility to *Mtb*. A cohort of 222 Chinese subjects with pulmonary TB was compared to 232 healthy controls and analyzed for six SNPs in exon 7 of the *MRC1* gene. In both frequency of genotype and allele, the G1186A site was significantly different between those with pulmonary TB compared to healthy controls (Zhang et al., 2012). These observations identify the G1186A mutation as a potential risk factor for the development of pulmonary TB. Speculatively, functional changes in MR due to the G1186A SNP may temper the pro-inflammatory or phagocytic outcomes of MR signaling upon *Mtb* infection, or alternatively, further inhibit phagosome maturation. To date, MR SNP that are linked to susceptibility to HIV and HIV/*Mtb* co-infections have not been described.

Polymorphisms in the *CLEC4E* gene that encodes Mincle have been associated with susceptibility to *Mtb*, although limited results to date suggest racial or other population differences. Mincle binding of mycobacterial TDM is strongly associated with TB granuloma formation. Four *CLEC4E* SNPs (rs10841845, rs10841847, rs10841856, and rs4620776) were identified in an African patient cohort of 416 confirmed TB cases and 405 healthy controls (Bowker et al., 2016). No significant association between these four SNP and TB disease was identified in this comparison (Bowker et al., 2016). A more recent study in a population from northern China further characterized these CLEC4E polymorphisms in association with pulmonary TB disease (Kabuye et al., 2019). Interestingly, studies in this population identified significantly increased variants of rs10841845 and rs10841847 in the 214 control patients compared to the 202 pulmonary TB patients. This suggests that functional polymorphism of CLEC4E may confer protection against pulmonary *Mtb* infection in this population (Kabuye et al., 2019). To date, *MRC1 and CLEC4E* SNP that are linked to risk for HIV infection or HIV/*Mtb* co-infections have not been described.

Human MBL, encoded by the *MBL2* gene, belongs to a family of soluble CLRs known as collectins that initiate complement activation. Polymorphisms of *MBL2* and the downstream signaling components which initiate the complement lectin pathway (Klassert et al., 2018), are associated with susceptibility to both pulmonary and extra-pulmonary TB across various populations (Denholm et al., 2010). In a cohort from the northeastern region of Brazil, *MBL2* promoter polymorphisms were linked with increased susceptibility to pulmonary TB. Interestingly, no significant correlations were observed between *MBL2* polymorphisms and extra pulmonary TB (da Cruz et al., 2013). Conversely, a relationship with extra-pulmonary TB was identified in a cohort of Chinese patients. In this analysis, MBL2 polymorphisms were found to correlate with a diagnosis of spinal TB (Zheng et al., 2018).

*MBL2* polymorphisms have also been associated with reduced levels of serum MBL, although the implications for *Mtb* susceptibility remain to be fully characterized (da Cruz et al., 2013). In one study of patients with ankylosing spondylitis (AS), a rheumatic disease characterized by low MBL levels, a significant correlation was identified between MBL deficiency and TB. The increased TB risk in those with low serum MBL was independent of either TNF therapy or latent infection in the patient medical history (Nisihara et al., 2018).

Susceptibility to HIV infection is also associated with MBL polymorphisms. In a 2011 study of a cohort from Southern Brazil, variant alleles of MBL were associated with increased susceptibility to HIV-1 infection (da Silva G. K. et al., 2011). Genotyping of HIV-infected patients of European and African descent in the cohort further revealed a difference in *MBL2* SNP frequencies and serum levels between ethnic groups. Interestingly, HIV-positive patients of European descent had low MBL serum levels whereas those of African descent had elevated MBL levels. The basis for this dichotomy is unknown and suggests that the full scope of immune responses regulated by *MBL2* are not yet understood.

In one report, *MBL2* alleles and genotypes were assessed in a Western European population for association with co-infection. Interestingly, HIV/TB dual disease was associated with low or non-producer alleles in comparisons with TB negative subjects including those with a positive HIV status (Garcia-Laorden et al., 2006). MBL deficiency may thus be associated with active TB in HIV co-infection. These findings demonstrate the broad spectrum of *MBL2* polymorphisms associated with HIV and TB disease across various populations, including those with co-infection.

## CLR Based Therapeutic Strategies

**Figure 2:**
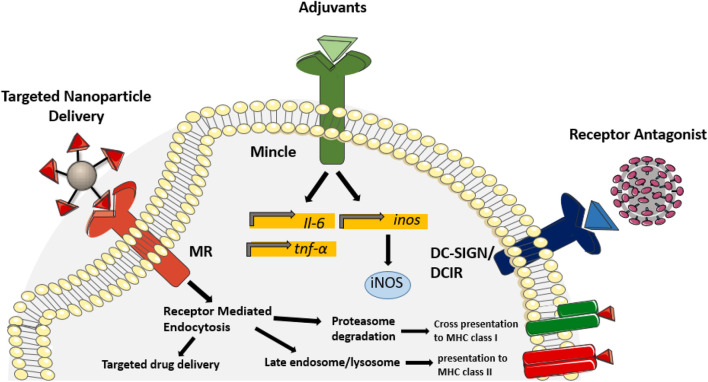
Therapeutic application of CLR ligands. In recent years, known CLR ligands have been used as therapeutic targets to improve uptake of drugs and vaccines, modulate protective immune responses and block receptor binding. Incorporating mannose derivatives on nanoparticles targets uptake through the mannose receptor, thus targeting antigen presentation pathways and improving drug delivery into alveolar macrophages. Due to the potent inflammatory response initiated by Mincle binding, derivatives of TDM have been incorporated as adjuvants to improve antigen-specific protective immunity during vaccination. Glycan derivatives have also been used as a mechanism to antagonize receptor binding of the HIV envelope, thus preventing viral transmission. Competitive inhibition of galectin-1, DC-SIGN and DCIR have shown efficacy in reducing HIV transmission to CD4^+^T cells. Targeting CLR signaling and binding has proven efficacious in various therapeutic applications and demonstrates the potential for CLR ligands in the treatment of infectious diseases.

The function of C-type lectin receptor signaling to shape innate and adaptive immune responses is exploited in recent approaches to develop novel therapeutics. CLR receptors are efficiently engaged by *Mtb* and HIV PAMPs and several of the resulting immune outcomes are characterized (Figure 2). As a result, mycobacterial and viral antigens that bind CLR have many therapeutic applications ranging from immune activation to cell targeted delivery. Competitive inhibition of CLR binding to viral particles also holds promise as a strategy for antiviral therapy.

### Vaccine Adjuvants

The glycosylation of both the mycobacterial cell wall and HIV virion is an important factor for recognition by CLRs and initiates downstream immune responses. Engagement of Mincle by TDM (Ishikawa et al., 2009) and phosphatidylinositol mannosides (Mosaiab et al., 2018) of mycobacteria induces robust pro-inflammatory cytokine response (IL-6 and TNF-α) and NO production *in vivo* (Ishikawa et al., 2009). Activation of Mincle signaling using TDM derivatives has been employed as an adjuvant strategy for TB vaccines (Desel et al., 2013; Decout et al., 2017) as well as for vaccines for unrelated pathogens (Azuma and Seya, 2001; Drummond et al., 2011). Of note, the adjuvant effect of TDM is dependent not only on Mincle, but also its co-receptor, MCL, constitutively expressed on myeloid cells (Tanne and Neyrolles, 2010; Miyake et al., 2013). Adjuvant outcomes could thus be tailored as needed for specific immune responses through strategies that target individual or multiple CLR pathways.

The choice of antibody subclass can also be modulated through CLR signaling. Generation of balanced IgG2a/IgG1 antibody subclasses specific to HIV is an important goal of DNA immunization. Activation of CLR signaling by HIV envelope was shown to regulate the antibody subclass response, as demonstrated by immunization of *Card9*^−/−^ mice (Hess et al., 2019). In contrast, antibody subclasses were not altered in *Myd88/Trif* ^−/−^ mice that lack functional TLR signaling (Hess et al., 2019). Application of CLR-targeting molecules for vaccine adjuvants is at an early stage of development. These early outcomes show promise to “fine-tune” the immune response as relevant to different microbial threats.

### Cell Targeted Delivery

Not only are CLRs capable of eliciting protective cytokine responses, but they also function as internalization receptors for delivery to antigen presentation pathways. Thus, the inclusion of CLR targets is increasingly employed to improve cellular uptake of drugs or vaccines. In a recent report, CLR-targeted delivery was shown to improve inhaled delivery of lipid nanoparticles loaded with rifampicin, a first line TB drug (Maretti et al., 2019). Decoration of lipid nanoparticles with novel mannose derivatives targeted the particle for uptake by MR. The result was improved efficiency of drug uptake by alveolar macrophages, the host cell for *Mtb* (Maretti et al., 2019).

A nanoparticle vaccine strategy that incorporated CLR targeted oligosaccharides has also been employed as a novel vaccination strategy for HIV. Co-delivery of mannose-enriched ligands that target DC-SIGN, with HIV peptides, increased the generation of antigen-specific CD8^+^ and CD4^+^T-cell responses (Climent et al., 2018). Targeting antigens to DCs through DC-SIGN has also been employed for TB vaccination. Conjugation of anti-DC-SIGN antibodies to the *Mtb* antigen Ag85B molecule elicited strong antigen-specific and polyfunctional (IFN-γ+, IL-2+, and TNF-α+) CD4^+^T cell responses (Velasquez et al., 2018). These strategies to target DC-SIGN may increase uptake and targeting to antigen presentation pathways as well as activate immune danger signals that promote development of polyfunctional T cell memory.

### Receptor Antagonists

The function of CLRs as potent endocytic receptors and innate immune activators is frequently exploited by pathogens for transmission and pathogenesis. Inhibition of these interactions thus provides opportunities for therapeutic intervention. Recently, blocking CLR-mediated viral transmission to naïve host cells was shown to serve as a host-directed therapeutic strategy for HIV. Galectin-1, a soluble CLR used by HIV for attachment and entry, may also serve as a target for anti-retroviral therapy. Treatment with carbohydrate lactoside compounds competitively inhibit the galectin-1 interaction with human CD4^+^T cells and HIV, and thus preventing attachment to uninfected cells (St-Pierre et al., 2012). Direct carbohydrate inhibitors are effective treatment strategies for CLRs with unique ligands. The carbohydrate recognition motifs shared by different CLRs are more challenging to target through inhibition strategies. For example, DC-SIGN shares ligand homology with Langerin. DC-SIGN is exploited by HIV to facilitate viral transmission while Langerin directs protective outcomes (de Witte et al., 2008; van der Vlist and Geijtenbeek, 2010).

One potential strategy is the use of glycomimetic technology to synthesize receptor targeted inhibitors specific for individual CLRs. Use of a glycomimetic to inhibit DC-SIGN binding reduced HIV trans-infection of T cells in human explant tissue models (Varga et al., 2014). At low μM concentration, these functionalized multivalent ligands also inhibited *in vitro* HIV trans-infection in isolated CD4^+^T cells (Varga, Sutkeviciute et al.). Blocking of other receptors which facilitate trans-infection of HIV, such as DCIR, have also shown efficacy in decreasing viral infection of CD4^+^T cells (Lambert et al., 2011). Use of glycomimetics and other CLR inhibitors thus have potential to reduce infections and may also have applications to modulate disease outcomes.

## Concluding Remarks

CLRs are essential PRR pathways of the innate immune response with diverse roles in immunity to *Mtb* and HIV. Our understanding of the roles of individual CLRs and the downstream immune responses in *Mtb* and HIV infection, and especially co-infection, is still evolving. Several CLRs bind *Mtb* ligands and activate innate, often pro-inflammatory, immune responses. There is redundancy in CLR function, as individual CLRs are generally dispensable with regards to TB disease outcomes in murine models. The roles for CLRs in HIV infection are less characterized due to the limitations for animal models. As currently understood, CLRs can generate protective anti-viral responses as well as serves as mechanisms for viral transmission. In the setting of co-infection, simultaneous signaling could drive convergent processes that differ from mono-infection and contribute to aggressive disease through mechanisms that are not yet understood. These overlapping mechanisms may also extend into other opportunistic infections and co-morbidities associated with *Mtb* or HIV infections, which may modulate innate immune signaling of CLRs. On-going applications that target CLR pathways demonstrate recognition of the translational potential for development of interventions for TB and HIV.

## Author Contributions

KN and JE both contributed conception and design of the review. KN wrote the first draft of the manuscript and developed the figure graphics. JE wrote sections of the manuscript and provided overall editing. All authors contributed to manuscript revision, read and approved the submitted version.

## Conflict of Interest

The authors declare that the research was conducted in the absence of any commercial or financial relationships that could be construed as a potential conflict of interest.
